# Supplementary material for: Causal Relationship between Aging and Anorexia Nervosa: A White-Matter-Microstructure-Mediated Mendelian Randomization Analysis
Source: Biomedicines. 2024 Aug 16;12(8):1874. doi: 10.3390/biomedicines12081874 (PMC11351342; doi:10.3390/biomedicines12081874)
Supplement: Supplementary file 1 [file biomedicines-12-01874-s001.zip › Supplementary Figure/Figure S8/Leaveoneout plot of MR analysis for TL to ubm-b-1724.pdf]

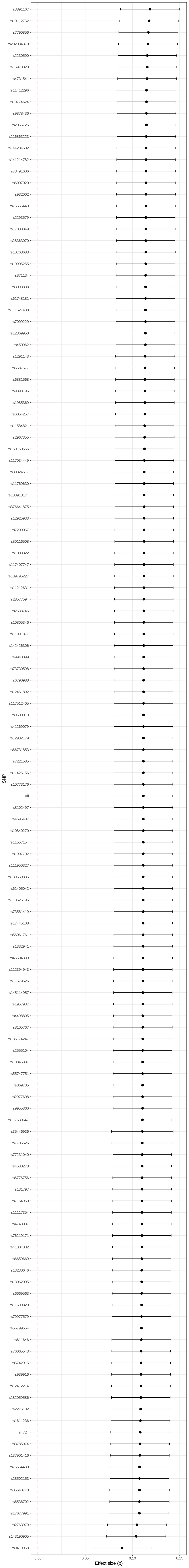
rs3891167
rs10112752
rs7790856
rs202034370
rs2230590
rs16978028
rs4731541
rs11412296
rs10774624
rs9878436
rs2056726
rs116863223
rs144204502
rs141214782
rs78491606
rs6007020
rs932002
rs76666449
rs2293579
rs17803849
rs28363070
rs10768683
rs10905255
rs871134
rs3093888
rs61748181
rs111527438
rs7099229
rs12369950
rs450962
rs1291143
rs6587577
rs6881568
rs9398196
rs1985369
rs6054257
rs11584821
rs2967355
rs150150565
rs117034449
rs80324517
rs11769630
rs188918174
rs376641875
rs12925933
rs7209057
rs80116508
rs1003322
rs117407747
rs139795227
rs11212631
rs28577594
rs2538745
rs10805346
rs11991877
rs142426306
rs9940099
rs73730598
rs6790988
rs12451892
rs117512405
rs9600019
rs41269079
rs12932179
rs66731853
rs7221585
rs11426156
rs10773176
All
rs8102497
rs4695407
rs10840270
rs11557154
rs1907702
rs111950327
rs139669835
rs61405042
rs113525195
rs73581419
rs17445108
rs56061761
rs1332941
rs45604339
rs112394943
rs11579626
rs145114957
rs1957937
rs4498805
rs8105767
rs185174247
rs2555104
rs10845387
rs55747751
rs869785
rs2977608
rs9955360
rs117630647
rs35446936
rs7705526
rs77231040
rs4530278
rs6776756
rs131797
rs7164950
rs11117354
rs4743037
rs76219171
rs41304832
rs6659669
rs13230646
rs13062095
rs6669563
rs11699829
rs79977579
rs56799554
rs611646
rs76065543
rs5742915
rs939916
rs12412214
rs182059586
rs2276182
rs1611236
rs4724
rs3785074
rs137901416
rs75664430
rs28502153
rs35640778
rs6536702
rs17677991
rs2763979
rs143190905
rs9419958
SNP
0.00
0.05
0.10
0.15
Effect size (b)
